# A Spectrophotometric Study on Light Attenuation Properties of Dental Bleaching Gels: Potential Relevance to Irradiation Parameters

**DOI:** 10.3390/dj8040137

**Published:** 2020-12-16

**Authors:** Eugenia Anagnostaki, Valina Mylona, Kyriaki Kosma, Steven Parker, Marianna Chala, Mark Cronshaw, Vasilis Dimitriou, Michael Tatarakis, Nektarios Papadogiannis, Edward Lynch, Martin Grootveld

**Affiliations:** 1 Leicester School of Pharmacy, De Montfort University, Gateway House, Leicester LE1 9BH, UK; vasiliki.mylona@my365.dmu.ac.uk (V.M.); steven.parker@my365.dmu.ac.uk (S.P.); Drmarkcronshaw@outlook.com (M.C.); edward.lynch@hotmail.com (E.L.); mgrootveld@dmu.ac.uk (M.G.); 2 Institute of Plasma Physics and Lasers, Hellenic Mediterranean University, Tria Monastiria, 74100 Rethymno, Greece; kosma@hmu.gr (K.K.); dimvasi@hmu.gr (V.D.); mictat@hmu.gr (M.T.); npapadogiannis@hmu.gr (N.P.); 3 Department of Surgical Sciences and Integrated Diagnostics, University of Genoa, 16132 Genoa, Italy; mariannachala@gmail.com; 4 School of Dental Medicine, University of Nevada Las Vegas, Las Vegas, NV 89154, USA

**Keywords:** dentistry, absorption, bleaching, laser, light, parameters, pulp, spectrophotometry, tooth colour, tooth whitening

## Abstract

Background: During in-office bleaching, appropriate light sources are applied in order to enhance the activity of the bleaching gels applied onto teeth. For this method to be effective, a high absorption of light within the gel is necessary. Variation in the light attenuation capability of the gel, the duration of application and light activation can contribute towards safety hazards associated with this procedure. Methods: In this study, seven different gels and hydrogen peroxide have been evaluated for their optical properties by means of spectrophotometry (440–1000 nm). The transmitted light spectrum was used to estimate the intensity loss for each gel. The mean intensity decreases observed were statistically analysed using an analysis of variance (ANOVA). Results: The five more-pigmented gels tested indicated a very similar intensity loss of around 80%, whereas the remaining two gels showed significantly less attenuation (predominantly, *p* < 10^−6^). Conclusions: Throughout the spectrum of wavelengths examined, and according to the underlying studies evaluated, five of the gels assessed demonstrated an attenuation high enough to possibly avoid overheating of the underlying enamel dentine and pulp. An evaluation of appropriate irradiation parameters is proposed.

## 1. Introduction

Dental bleaching is a widely performed therapy, with the primary goal to achieve brighter and whiter tooth colouration. During bleaching, a specific gel is applied on a previously cleansed and polished tooth surface [1]. The main active ingredient of bleaching gels is hydrogen peroxide (H_2_O_2_) or carbamide-peroxide, the latter representing a 1:1 additional complex of H_2_O_2_ with urea [2]. The procedures utilised can be performed either as home-bleaching or in-office techniques. According to Auschill et al., a single in-office bleaching session (i.e., a 3 × 15 min application of gel) corresponds to, or gives similar results to, one week of home-bleaching applications (7 × 8 h). The concentrations of H_2_O_2_ (or equivalent) used for the former is about 35% (*w*/*w*) and 5% (*w*/*w*) for the latter [3]. The use of H_2_O_2_ concentrations above 6% (*w*/*v*) is only allowed for tooth-whitening episodes following medical indications within the EU [4].

In order to further shorten the interaction time of the gel on dental tissue, it can be activated by the supporting application of light [5]. The primary objective of applying a light source is the selective absorption by pigmented agents present in bleaching gels to achieve a photothermal containment within the gel, which increases the rate of the chemical fragmentation of H_2_O_2_ to generate highly bleaching-active hydroxyl radicals (^●^OH) (Equation (1)).

H_2_O_2_ + *hν* → 2^●^OH (1)

where *h* is Plank’s constant, and *ν* is the frequency of light.

Various kinds of incandescent lamps, light-emitting diodes (LEDs) and lasers have been described in the scientific literature, with lasers gaining more attention in recent years [1,6]. Different light sources can have markedly different physical characteristics: a laser is typically represented as a stream of single-wavelength photons in temporal and spatial phase coherence, with high directionality and monochromaticity; in comparison with a broader bandwidth, noncoherent, low-directional light source, such as an LED, a laser is a much more intense source of energy [7].

Light passing from one medium to another can undergo reflection, remission, refraction, scattering, absorption and onward transmission in amounts which depend on the material properties and the light characteristics, such as wavelength and intensity [1]. For an efficient activation of the bleaching gel, a high absorption coefficient is the relevant influencing factor; any heat produced as a result of gel irradiation should be ideally kept within the confines of the gel to avoid any overheating of the underlying vital dental tissues. Therefore, specific wavelength-appropriate absorbers are added to the gel, and as such, some gels are best-suited for applications employing a matched activation source [8].

Shorter wavelengths of light (400–600 nm) are associated with higher photon energy than the commonly employed longer wavelengths (800–1000 nm). Potentially, this may give rise to a higher level of heat induced in the applied gel; the significance of the adequate gel applied and an adequate attenuation potential is important to mitigate the possible onward transmission of photons through the enamel and dentine and on to the pulp chamber. It is acknowledged that an unwanted temperature rise in the tooth structure may influence the onward transmission [8,9,10,11]. Of significance, the alignment of the underlying enamel prisms and dentinal tubules can act as a photo-optical conduit towards the pulp [11,12].

It is generally considered that the thermal damage threshold of the pulp is reached through a temperature elevation of 5.5 °C before encountering a progressive loss of pulp vitality [13]. Moreover, Eriksson et al. evaluated the duration of tolerable temperature increase and reported that an increase of 5 °C for only one minute represented a critical threshold, beyond which the health of the vital pulp tissue may be imperilled [14].

In this experimental study, the attenuation properties of various gels were investigated to evaluate their light intensity loss through a spectrophotometric analysis. The transmitted light intensity per different wavelengths throughout the range sampled is recorded, since this is the moiety that passes through the bleaching gel towards the tooth surface and then, after similar interactive phenomena (reflection, remission, refraction, scattering, absorption and transmission), may eventually reach the tooth pulp.

Knowledge of the light attenuation is crucial for the calculation of laser activation parameters, and currently, no clear recommendations exist on laser activation parameters during bleaching. The spectrophotometric measurement of the fraction of light transmitted through the samples is a reliable method commonly used in different studies [15,16]. To the best of our knowledge, until now, there has been no extensive comparative study on more than two different bleaching gels relating absorption by the gel-to-light attenuation at the tooth surface. Thus, in the present study, the intensity loss of light passing through seven bleaching gels was evaluated via a spectrophotometric approach. The null hypothesis was that all dental bleaching gels and the H_2_O_2_ evaluated, exerted the same light attenuation properties, irrespective of the visible/near-infrared region wavelength applied.

## 2. Materials and Methods

### 2.1. Tested Components

The following bleaching gels currently employed in clinical dental therapy were tested as indicated in Table 1. Additionally, a solution of 30% (*w*/*v*) H_2_O_2_ in water was tested as well, as this is the common ingredient among all bleaching gels.

The ingredients of each bleaching gel investigated are declared in the respective Material Safety Data Sheets (MSDS) [17,18,19,20,21,22,23].

### 2.2. Light Sources

A broadband emission halogen lamp light source (TekLight, MilleLuce, Santa Cruz, CA, USA) was used without an internal infrared (IR) cut-off filter. The halogen emission spectrum ranged from 400 to 1000 nm.

### 2.3. Testing Procedure

**Figure 1:**
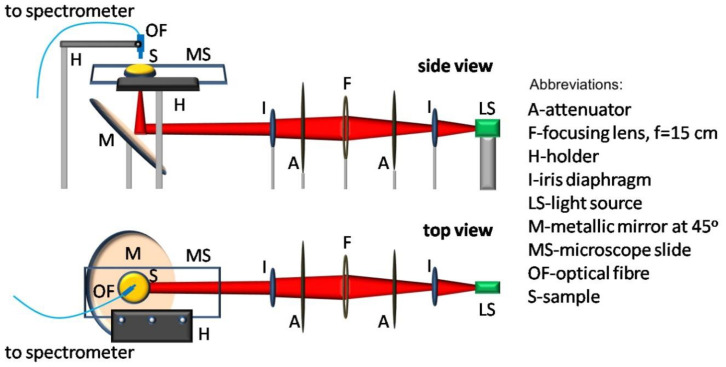
Experimental set-up for the spectrophotometry measurements performed on dental bleaching gels.

The complete experimental procedure was performed at a stable room temperature of 18 °C on a vibration-free optical table at the laser laboratory of the Institute for Plasma Physics and Lasers of the Hellenic Mediterranean University Rethymno, Greece. The experimental set-up and the optical elements used for performing the spectral measurements are explained in Figure 1. The central part of the output light from the source (LS) (halogen lamp), selected by an iris diaphragm (I), passed through an attenuator (A) and a biconvex lens (F) (focal length *f* = 15 cm) and was directed towards the microscope slide. Before reaching the metallic mirror (M), the light beam passed through a second attenuator (A) and an iris diaphragm (I), which further controlled the intensity and the divergence of the beam to be focused on the gel under study.

All bleaching gels tested were freshly mixed following each manufacturer’s recommendations and applied on a glass microscope slide, on which a standardised metal ring (1-mm height, 11-mm inside diameter and 0.95-mm^2^ area) serving as a gel holder was previously attached. The excess gel was removed with a spatula within the vicinity of the ring, so that the gel layer thickness (1 mm), similar to that applied onto the tooth, remained consistent for all samples. The microscope slide was mounted on a holder placed parallel to the optical table, as shown in Figure 1. The slide holder allowed for a relatively easy movement and a replacement of the different glasses containing the gels to be examined in this manner at a standardised position at a distance of 2 cm from the mirror (M).

After passing through the microscope slide carrying the gel sample, the light was transmitted to the optical sensor fibre (QP100-2-VIS-BX, Ocean Optics, Largo, FL, USA) and the linked high-resolution spectrophotometer (HR4000CG-UV-NIR, Ocean Optics, Largo, FL, USA) with a wavelength selectivity of 400–1000 nm for analysis. To collect the transmitted light, the tip of the sensor fibre was mounted perpendicular to the glass slides and above the rings containing the analytical samples. The transmitted spectrum was recorded firstly for the glass alone and, secondly, for the gel on the glass at an integration time of 10 s. The mean of *n* = 3 replicate measurements was obtained. The results were then transferred to a PC, for the purpose of computing the light intensity modifications, which were plotted as a function of the wavelength and visualised through “Oceanview” software (Ocean Optics, Largo, FL, USA).

The same procedure was applied on a solution of 30% (*w*/*w*) H_2_O_2_, as this was the common ingredient amongst all bleaching gels evaluated here.

### 2.4. Data Collection

Attenuation was measured by an evaluation of the light intensity losses through each product tested expressed relative to those observed in the absence of the respective product, and expressed as a %. This was computed according to the formula provided in Equation (2), where I = intensity.

100[(Ι_(gel on glass)_ − Ι_(glass alone)_)/Ι_(glass alone)_] (2)

Photo-optical data for each gel were transferred and further analysed using the “GraphPad Prism” software module, Version 9.0.0 for a Mac (GraphPad Software, San Diego, CA, USA; www.graphpad.com). The proportional intensity change values were plotted versus the wavelength to generate a range of visible absorption spectra, and this change was attributed as attenuation of the light that underwent absorption, reflection and scattering through each gel.

### 2.5. Statistical Analysis: Experimental Design, Power Calculations, Transformations and Satisfaction of Normality and Homoscedasticity Assumptions

The mean proportional intensity changes observed were statistically analysed using an analysis of variance (ANOVA)-based experimental design, which was classified as a two-factor system with treatments (*n* = 7, plus an H_2_O_2_ control) and selected wavelengths at which the measurements were made (445, 532, 880, 940, 970 and 980 nm) being fixed qualitative effects at 8 and 6 levels, respectively. Three replicate determinations were made for each treatment-wavelength combination. The mathematical model for this design is shown in Equation (3), where µ represents the null mean value in the absence of all sources of variation, and T*_i_*, W*_j_*, TW*_ij_* and e*_ijk_* represent the “between-treatments” and “between-wavelengths” and treatments × wavelengths first-order interaction effects (all fixed effects) and unexplained sampling error sources of variation, respectively. The term y*_ijk_* represents the monitored response variable, i.e., proportional light intensity change. The interaction effect was included so that the differential responses of each treatment applied could be explored across the full wavelength range. Statistical analysis was performed using *XLSTAT2020* software (Addinsoft, New York, NY, USA; www.xlstat.com).

y*_ijk_* = µ + T*_i_* + W*_j_* + TW*_IJ_* + e*_ijk_* (3)

The probability value *p* for significance was set to <0.05.

Although no pre-experimental sample size calculation was performed, we elected to determine such values retrospectively following the full completion of spectral data acquisition. For this purpose, we firstly estimated those required for two sample comparisons, the computations involved being customary ones based on normal distributions of experimental sample groups. Using our original (untransformed) dataset, these estimated sample size values for the default significance and power values of 0.05 and 0.80, and with a residual standard deviation value determined from the error mean square parameter of our two-factor ANOVA model conducted, were 9, 11, 14, 25 and 56 for mean (absolute) intensity losses of 0.95, 0.90, 0.80, 0.60 and 0.40. Therefore, we concluded that the sample size of *n* = 18 (including 3 replicates at each wavelength for each treatment applied) for our treatment groups had the power to detect a mean difference lying between 0.60 and 0.80. Similarly, a sample size of *n* = 24 was found to be sufficient to detect a mean “between-wavelength” difference of as low as ca. 0.60.

Secondly, we employed the WebPower effect size calculator for two-way ANOVA models and found that estimated optimal sample sizes for treatments, wavelengths and the treatment × wavelength first-order interaction effects were 6.12, 6.22 and 6.75, respectively, for significance and power levels of 0.05 and 0.80, respectively, and therefore, those used for our experimental model were again found to be more than sufficient for statistical analysis purposes.

Following application of the above ANOVA strategy for statistical analysis, however, plots of model residuals against model-predicted values revealed that the dataset deviated significantly from following a normal distribution (Shapiro-Wilk test) and nor was it homoscedastic (Levene’s test). Therefore, primarily, we applied a logarithmic (log_10_) transformation to the entire dataset, since such a process is known to approximately normalise data that follow non-normal distributions and, also, homogenise intrasample variances; both of these criteria are critical assumptions for the performance of parametric statistical analyses such as those featured in ANOVA models. However, unfortunately, this procedure failed to normalise the above model residuals and nor did it render their variances homogenous. Therefore, these transformed data were then subjected to the Box-Cox transformation, which did successfully normalise the ANOVA residual dataset when analysed again in this manner and, also, homogenised the variance of these model residuals.

In view of the predominantly negative values of the proportional intensity loss data (x) values acquired, the first transformation applied was log_10_[−(x−10)]; transformed values arising therefrom were then secondly transformed using the Box-Cox transformation.

We also employed the Shapiro-Wilk test to check the normality of the 3 replicate measurements made for each wavelength and each treatment. This test did not reveal any deviations from normality for all sets of *n* = 3 replicates.

Following statistical analysis of the normalised, homoscedastic Box-Cox- and log_10_-transformed dataset with our ANOVA model, Bonferroni’s *post-hoc* ANOVA test was employed to determine the significance of individual mean differences “between treatments” and “between wavelengths”.

## 3. Results

**Figure 2:**
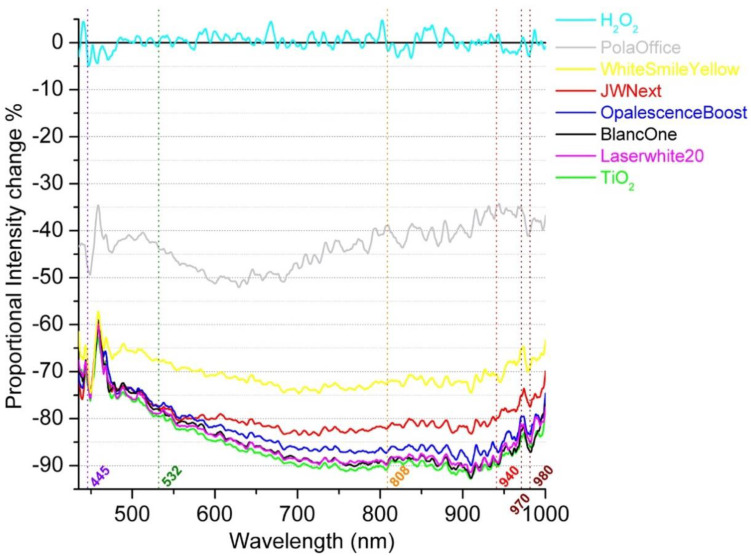
Light attenuation curves (average spectra) of all bleaching gels and H_2_O_2_ analysed.

Attenuation spectra of the various bleaching gels and a 30% *w*/*w* H_2_O_2_ solution in the 440–1000-nm region through 1 mm of gel are shown in Figure 2.

The light source used was a halogen lamp. The abscissa axes represent the range of laser wavelengths that are commercially available in dentistry.

It was observed that five gels (JW Next, Laserwhite20, BlancOne, OpalescenceBoost and TiO_2_) were presenting similar attenuation behaviours in intensity changes, while WhitesmileYellow was showing a slightly lower change. The lowest intensity change was presented by the PolaOffice+ gel. The H_2_O_2_ control solution was transparent in visible and NIR light, as expected and assuming the background electronic noise of the detector that is represented in all samples. In addition, the oscillations at the edges of the spectrum appear to arise from electronic noise.

**Figure 3:**
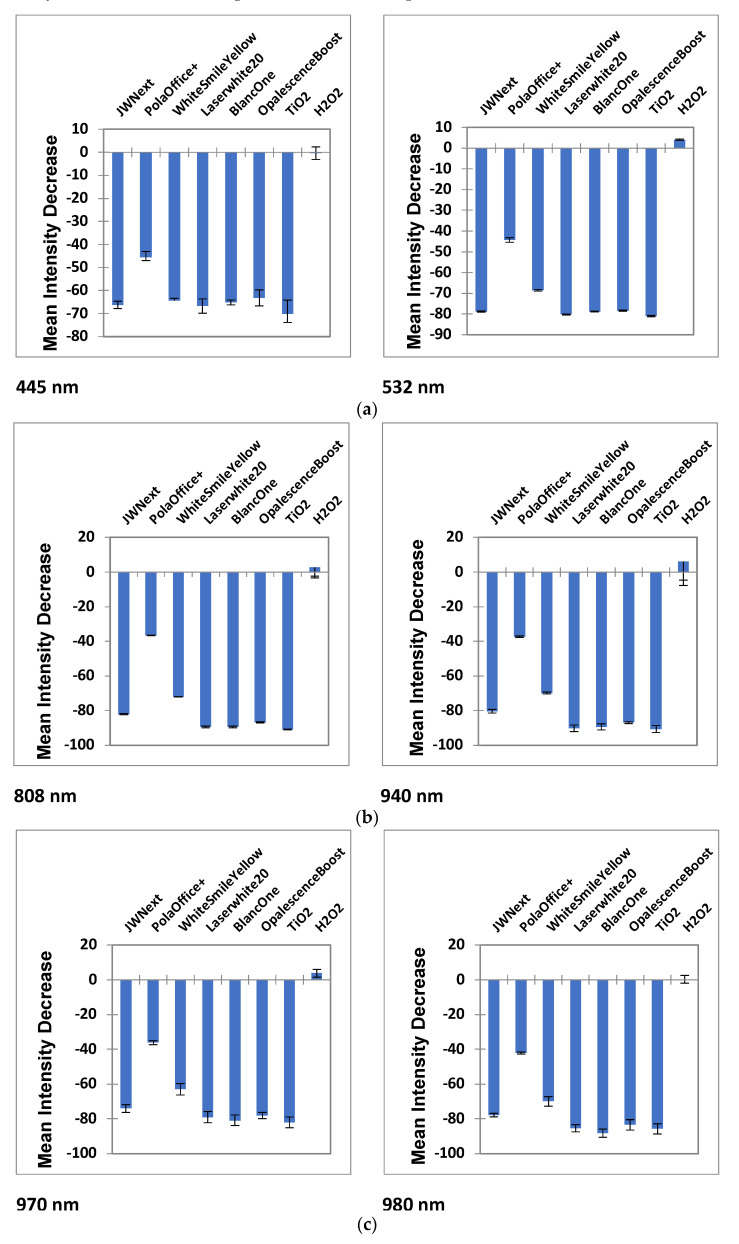
(**a**) Mean ± 95% confidence intervals of the decrease in proportional intensities observed at 445 and 532 nm for the products investigated. (**b**) Mean ± 95% confidence intervals of the decrease in proportional intensities observed at 808 and 940 nm for the products investigated. (**c**) Mean ± 95% confidence intervals of the decrease in proportional intensities observed at 970 and 980 nm for the products investigated.

Additionally, mean ± 95% confidence intervals (CI) of the decrease in intensities observed at 445, 532, 808, 940, 970 and 980 nm for the products investigated are shown in Figure 3a–c. The five gels (JW Next, Laserwhite20, BlancOne, OpalescenceBoost and TiO_2_) interacted more with NIR wavelengths (808, 940, 970 and 980 nm). In the visible region (445 and 532 nm), this high attenuation decreased. PolaOffice+ showed a range of proportional intensity change approximately between 37% and 44% for all wavelengths, while WhitesmileYellow showed changes between 64% and 73%, respectively. As before, H_2_O_2_ was presented to be transparent.

**Figure 4:**
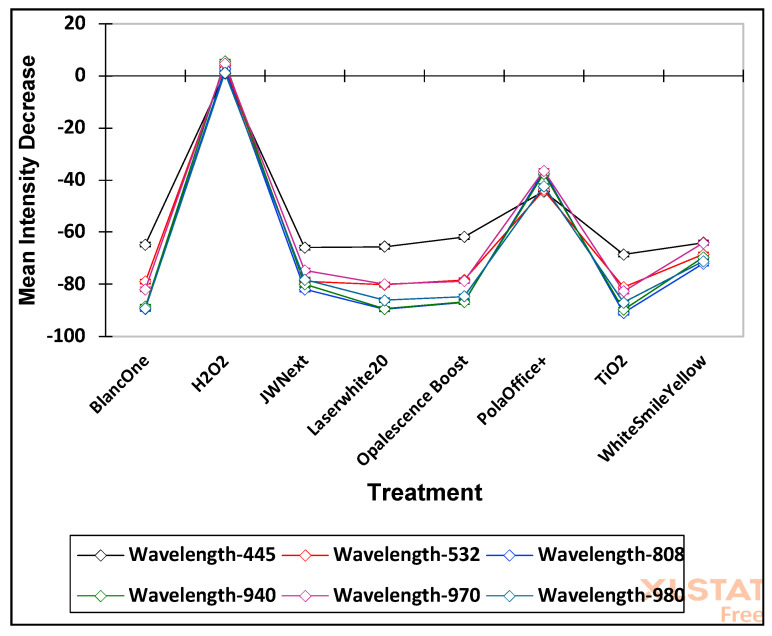
Plots of mean intensity decrease responses from each of the 7 products and H_2_O_2_ control treatments evaluated at wavelengths of 445, 532, 808, 940, 970 and 980 nm.

Moreover, ANOVA of the Box-Cox-normalised and log_10_-transformed mean proportional intensity loss dataset revealed that differences observed in the light intensity decrease values “between treatments” and “between wavelengths” were astoundingly statistically significant (*p* = 6.27 × 10^−157^ and 1.53 × 10^−71^, respectively). However, the first-order “treatments × wavelengths” effect was also extremely significant (*p* = 3.32 × 10^−53^), and this observation provides powerful evidence for differential wavelength dependencies in the mean proportional intensity decreases observed. This effect is most readily visible in Figure 4.

The application of Bonferroni’s *post-hoc* ANOVA test revealed that the statistically significant difference between gels (in intensity loss) is indicated from the highest to lowest values as follows: TiO_2_ > BlancOne ≈ LaserWhite20 > OpalescenceBoost > JWNext > WhiteSmileYellow > PolaOffice+ > H_2_O_2_. All the differences indicated had *p* values lower than 10^−6^, with the exception of the TiO_2_ *versus* BlancOne comparison difference, which had a *p* value of 5.77 × 10^−6^, and that between BlancOne and LaserWhite20, which was not at all significant. As demonstrated in Figure 3 and Figure 4, H_2_O_2_ clearly showed extremely significantly lower attenuation values than all the bleaching gels examined.

Similarly, for wavelengths, the greatest intensity loss was observed at 808 nm and the lowest at 445 nm.

**Figure 5:**
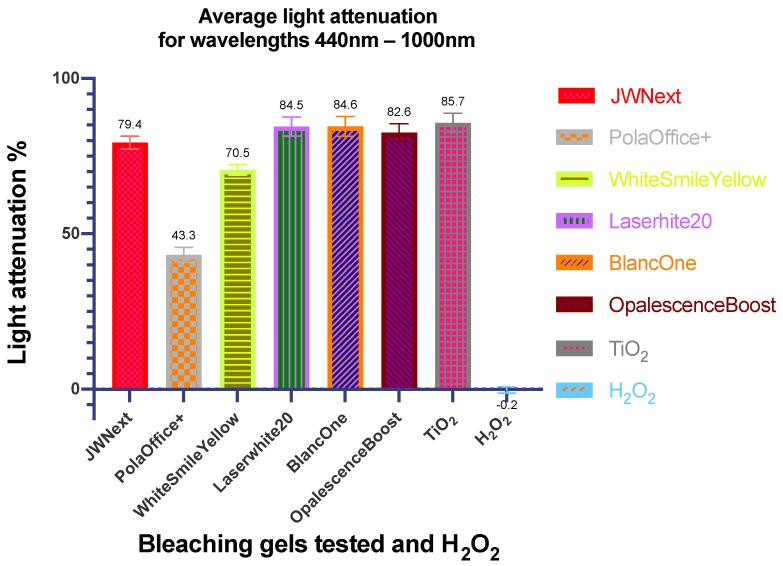
Bar diagram showing the mean ± SD values for light attenuations observed across all wavelengths tested by the bleaching gel products and 30% (*w*/*w*) H_2_O_2_ control evaluated.

Finally, the respective mean attenuation values ± SD for the total spectrophotometric range of 440–1000 nm are shown in Figure 5.

It was clear that JW Next, Laserwhite20, BlancOne, OpalescenceBoost and TiO_2_ had notable interactions with light, while the WhitesmileYellow product exhibited a slight difference. PolaOffice+ presented the lowest attenuation percentage from all gels, and light could pass through H_2_O_2_ without any change, as expected.

## 4. Discussion

In the present study, the proportional intensity loss of light (440–1000 nm range) passing through various bleaching gels was evaluated; this wavelength range represents that of laser systems most commonly employed in dentistry. The analysis of H_2_O_2_ was performed because all gels currently available contain mainly water and H_2_O_2_, and it was therefore employed as a control. Specifically, the H_2_O_2_ content in gels used for in-office dental bleaching usually lies within the 28–38% (*w*/*w*) range.

The extremely significant (*p* = 3.32 × 10^−53^) “treatments × wavelengths” effect provides powerful evidence for differential wavelength dependencies in the mean intensity decreases observed. This effect is most readily visible in Figure 4, which shows that, although some of the products (BlancOne, JW Next, LaserWhite20, Opalescence Boost and TiO_2_) behave relatively similarly across all wavelengths, all exhibit much less intensity loss than the other products tested at a wavelength of 445 nm. Therefore, this is one of the major reasons for the significant interaction effects observed.

The average intensity loss of most gels (BlancOne, JW Next, LaserWhite20, Opalescence Boost and TiO_2_) within the wavelength range examined (440–1000 nm) was between 79% and 86%. Even with different gel colours, which ranged from orange, dark red, light red or even purple, the light attenuation was very similar for all products assessed throughout the spectral region examined. This finding implies that not only the defined pigment chromophores contained in the gel but, perhaps, one of the other additives, which they all have in common, cause similarities in light attenuation properties. Only PolaOffice+ and WhitesmileYellow differ notably from the others examined, showing a highly significantly lower light attenuation of 43% and 71%, respectively (*p* < 10^−6^). This might be explained by the fact that these two gels contain virtually no absorbent pigment. Concerning the TiO_2_ gel, reflection might simultaneously occur in a range of directions, and this represents a component of the light attenuation, along with absorption and backscattering. Although this product does not contain a pigment and, hence, does not absorb visible light, it does reflect, and therefore, less light will penetrate the sensor. Added TiO_2_ serves as a visible light-activating photocatalytic system [23,24].

Goharkhay et al. stated that there is an exponential increase in the laser beam attenuation with transmission depth when expressed relative to the absorption coefficient of the gel. According to the choice of photo-absorber, the absorption coefficient can vary, as does the wavelength itself [23]. Specifically, based on the findings of the present study, the 445-nm wavelength exhibited the least light intensity loss for all products examined. Moreover, there is a correlation between the temperature increase within the gel and pulp chamber and the absorption coefficient of the bleaching gel [23]. According to Buchalla and Attin [8], the absorption of blue light is increased by the orange-red colour of carotene. Red and infrared light absorption can be increased by the addition of small silica particles of nm or lower μm dimensions, which gives these gels a blue colouration [8].

The active component in the bleaching gel, specifically H_2_O_2_, contributes to the overall process through a series of induced gel-heating episodes, with its decomposition arising from photoexcitation of the parent molecule. Additionally, photochemical actions may be exerted by product pigments [24].

The suggested efficacious temperature of a bleaching agent when applied on vital teeth ranges from between 46 to 60 °C and is equivalent to a temperature rise of approximately 15–30 °C within the gel [25]. However, the authors of the present work advise that such upper limits should be used with caution or at least until more studies show consistent and reliable results. The published data refers to the consequence of a temperature rise where the rate of chemical reaction in the gel may double with just a 10 °C increase and how that may be maximised and contained within the gel through the employment of wavelength-specific high-absorbing pigments [26]. 

Moreover, specific substances, such as carotene and TiO_2_, when irradiated can destabilise H_2_O_2_ and/or cause an imbalance in bleaching gel pH values through physicochemical interactions. These actions give rise to reactive oxygen radical generation [24]. However, De Moor et al. questioned whether the dyes added for photoactivation of the gel with commonly employed laser systems are helpful in directly activating the bleaching gel via photochemical actions [26].

Following the prior generation of the aggressively reactive, tooth-whitening ^●^OH radical (Equation (1)), the mechanism of H_2_O_2_ degradation involves two further steps—the first involving the reaction of the ^●^OH radical product with further H_2_O_2_ (Equation (4)) and the second reaction of ^●^OOH generated in Equation (2) with further H_2_O_2_ (Equation (5)) [2].

^●^OH + H_2_O_2_ → ^●^OOH + H_2_O (4)

^●^OOH + H_2_O_2_ → 2^●^OH + H_2_O + O_2_ (5)

Since the photon energy of a specific wavelength can be easily calculated with the Plank-Einstein equation (E = hν or E = hc/λ, where E represents photon energy, h is Planck’s constant, ν is the energy frequency, c is the speed of light in a vacuum and λ is the photon’s wavelength), a direct light-induced dissociation can only be achieved by lasers of wavelengths <614 nm. The molecular bond dissociation energy of H_2_O_2_ is 2.02 eV [24,27].

In view of the above facts, it appears that laser-activated bleaching predominantly involves photothermal reactions. Hence, the heat transmitted to the dental pulp needs to be controlled, both to optimise the speed of reaction but, also, to safeguard against adverse post-bleaching sensitivity reactions.

For all wavelengths investigated in previous corresponding studies, light is transmitted through the bleaching gel in combination with a heating process and relative to the gel thickness; this could give rise to tooth sensitivity. This is the most intimidating adverse effect of bleaching procedures [28]. The mechanisms associated with post-bleaching sensitivity may include pulpal inflammation as a reaction to hyperthermia, a direct phototoxic effect, or chemical hypersensitivity to the reaction products generated from the decomposition of peroxide gels and possible tooth surface desiccation. Additionally, Khan et al. investigated the cellular mechanisms underlying near-infrared (NIR)-induced phototoxicity and found an association with surface temperature and reactive oxygen species (ROS) activity [29]. According to Caviedes-Bucheli et al., a further plausible explanation is that light- and laser-activated bleaching may increase the expression of substance P, a neuropeptide, in human dental pulp tissue [30].

Since, currently, there are no studies focused on the effect of light passing through the gel to the enamel towards the dentine and pulp, it is necessary to calculate and derive conclusions from the landmark studies in the field of laser–dental tissue interactions [31,32,33,34]. Due to the optical properties of enamel and dentine, the following has to be considered. With reference to the enamel, assuming a buccal layer of 1–1.3 mm [25], visible or NIR irradiation can penetrate and lead to heating predominantly of the dentinoenamel junction (DEJ) region and the underlying layers and, eventually, harm the pulp vitality [32,33]. With reference to the light passing through dentine, according to the study by Kreisler et al., pulp viability can be maintained where there exists a mean minimal dentine layer of 2 mm with the power output limited to 1-Watt maximum irradiation for 10 s. However, in this work, the authors were irradiating human dentine (roots) with a 400-μm fibre at a distance of 1 mm [34]. Calculating the power density for these parameters (assuming a tip-to-tissue distance of 1 mm and a beam divergence of 12°), the result was 187 W/cm^2^, and for an irradiation time of 10 s, the fluence was 1870 J/cm^2^.

To calculate the maximum acceptable fluence for the pulp, a thermal relaxation time (TRT) has to be also considered, as this dictates that smaller targets cool faster [35]. The thermal relaxation time (TRT) is dependent on the square of the diameter (d) of the irradiated tissue:

TRT = d^2^/16α (6)

α is the thermal diffusivity, which is constant and related to penetration depth and tissue composition (Equation (6)) [36].

Application of the TRT (Equation (6)) with the tip diameter used in the Kreisler study [34] where the pulp was point-irradiated, d^2^ is (400^2^μm) = 0.0016 cm, whereas, with a flat-top handpiece, frequently used in dentistry, irradiation d^2^ is approximately 1^2^ cm = 1 cm. Assuming identical tissue and consequent thermal diffusivity, the thermal relaxation time (TRT) with a flat-top handpiece irradiation is 625 times longer.

With the use of a single-tooth bleaching tip with a diameter of 6 mm [23] at a distance of 10 mm from the target and with a beam divergence of 12°, the irradiation d^2^ is also 1^2^ cm = 1 cm. Thus, the “point-irradiated” pulp will accept 1870 J/cm^2^, whereas the flat-top handpiece or Gaussian single-toothed handpiece irradiated pulp will accept (1870/625) = 2.99 J/cm^2^.

The predominance of beam profiles with commercially available lasers in dentistry is a Gaussian distribution [37]. The risk is that, despite the average power value of the beam, there is a doubling of this at the centre of the applied beam, together with a reduction in applied irradiance at the periphery equal to 13.5% of the peak value [38]. Consequently, beams with Gaussian distribution might carry significant risk related to the peak irradiance. Conversely, the absence of low fluence periphery and steeper edge transitions in flat-top beams allow for a smaller heat-affected zone and more efficient delivery of energy [39]. Accordingly, a flat-top uniform energy distribution might provide a more accurate value of fluence and irradiation.

Based on the calculations above, it can be estimated that, after having passed through the bleaching gel layer, the fluence should not exceed 2.99 J/cm^2^. If the light, after passing through the bleaching gel, undergoes an intensity loss, as shown here, of approximately 80%, that means that the maximum permissible fluence for laser-activated bleaching should be 14.96 J/cm^2^. The latter coincided with the fluence applied in the investigation carried out by Sari et al., who recorded a pulp temperature increase of 2.61 °C. The parameters applied in this study were a wavelength of 830 nm, bleaching gel WhitenessRed HP 35%, and 13.67 J/cm^2^ fluence [40].

In a further comparison with existing studies, Al-Karadaghi et al. [16] reported the application of a 940 and 980-nm diode laser-assisted bleaching gel (LaserWhite20) whitening with a fluence of 43.2 and 72.3 J/cm^2^, respectively. This group of investigators, until now, performed the only spectrophotometric measurement of a bleaching gel (LaserWhite20) and gave a reference of absorption of 2.634 arbitrary units (AU) for the 940-nm laser versus 2.624 AU for the 980-nm laser [16]. In the present investigation, the same bleaching gel (Laserwhite20) shows an average optical transmission loss of 84.5%. Particularly, at 940 nm, it is 88.7% versus 88.5% at 980 nm.

Since there are inconsistencies in previous studies [8,16,40], at present, there is no consensus regarding the safety of the materials and parameters currently used. There is a need to consider the limitations of the spectrophotometric experimental set-up insomuch as extrapolation to a wider interpretation of the enhanced chemical effects, as well as to conclude or recommend specific clinical parameters. However, the intent of the paper is to present the investigation as an example of the many differing parameters for a clinician to consider; some discussion has been made to identify some of these issues, without necessarily attempting a direct consequence of the in vitro investigation. The extrapolation to in vivo conditions is to be handled with care, as too many factors apply by way of pulp blood flow, tooth condition, size, age, etc. [41,42].

When performed correctly, tooth bleaching is a safe technique, even if it can have some undesirable consequences, such as temporary postoperative sensitivity. Such effects can appear due to a response to the penetration of free radicals towards the dentine and pulp or by a direct activation of neuronal receptors caused by H_2_O_2_, as well as provoked by dehydration or overheating of the pulp tissue [26,28,43]. Effects on enamel surface morphology are also reported among the drawbacks of dental bleaching [44,45,46]. In vitro experiments indicate detrimental effects, whereas in vivo studies indicate less dramatic results. A systematic review by Attin et al. concluded that, if the study design simulates in vivo conditions as close as possible, the risk of enamel microhardness reduction is decreased [46]. Clinically, the short application time and use of noncorrosive, pH-neutral or alkaline gels is recommended [47]. Overall, side effects can be minimised with modern desensitising materials [48,49] and techniques [50,51]. Notwithstanding, a general review of the safety of tooth whitening has been published and it is reassuring that the number of clinically reported adverse effects or incidents after bleaching is relatively low [52,53,54].

## 5. Conclusions

This study focused on the effects of different bleaching gels on the loss of light intensity during a representative irradiation process in the wavelength range from 440 to 1000 nm. Through this, the clinician will be able to choose some appropriate bleaching gels for laser or light-activated bleaching.

The two, more transparent bleaching gels investigated in this spectrophotometric study were less effective in attenuation values and seem to be less suitable for light-activated bleaching. The remaining five gels show an attenuation high enough to help avoid a possible overheating of the underlying enamel dentine and pulp.

Further studies on pulp and bleaching gel temperature increases during laser-activated bleaching with the same gels and with the calculated safe parameters should be performed in order to obtain a consensus on the safety and effectiveness of this procedure.

## Figures and Tables

**Table 1:** Bleaching gels investigated and their corresponding active ingredients, contents, mixing procedure and manufacturer.

Bleaching Gel	Manufacturer	Active Ingredient (*w*/*w*%)	Colour	Mixing/Procedure
JW Next	Heydent/Kaufering, Germany Branded also for DoctorSmile/Elexxion	H_2_O_2_ (35%)	Red	Double-cartridge syringe/self-mixing tips
Pola Office+	SDI, Bayswater, Victoria, Australia	H_2_O_2_ (37.5%)	Blue-Transparent	Double-cartridge syringe/self-mixing tips
Powerwhitening YF WhitesmileYellow	Whitesmile, Birkenau, Germany	H_2_O_2_ (32%)	Yellow	Double-cartridge syringe/self-mixing tips
LaserWhite 20	Biolase, Irvine, CA, USA	H_2_O_2_ (38%)	Lavender	Syringe-to-syringe mixing
BlancOne Ultra	IDS Spa, Savona, Italy	H_2_O_2_ (29%)	Orange	Powder provided in a mixing container, H_2_O_2_ solution has to be added and mixed by hand
Opalescence Boost	Ultradent, South Jordan, UT, USA	H_2_O_2_ (38%)	Dark Red	Syringe-to-syringe mixing
JW TiO_2_	Heydent, Kaufering, Germany	H_2_O_2_ (30%)TiO_2_ (3.78% w/v)	White	Powder provided in a mixing container, H_2_O_2_ solution has to be added and mixed by hand

## References

[1] De Moor R.J.G., Verheyen J., Diachuk A., Verheyen P., Meire M.A., De Coster P.J., Keulemans F., De Bruyne M., Walsh L.J. (2015). Insight in the chemistry of laser-activated dental bleaching. Sci. World J..

[2] Grootveld M., Lynch E., Page G., Chan W., Percival B., Anagnostaki E., Mylona V., Bordin-Aykroyd S., Grootveld K.L. (2020). Potential Advantages of Peroxoborates and Their Ester Adducts Over Hydrogen Peroxide as Therapeutic Agents in Oral Healthcare Products: Chemical/Biochemical Reactivity Considerations In Vitro, Ex Vivo And In Vivo. Dent. J..

[3] Auschill T.M., Hellwig E., Schmidale S., Sculean A., Arweiler N.B. (2005). Efficacy, side-effects and patients’ acceptance of different bleaching techniques (OTC, in-office, at-home). Oper. Dent..

[4] Council Directive 2011/84/EU. https://eur-lex.europa.eu/LexUriServ/LexUriServ.do?uri=OJ:L:2011:283:0036:0038:en:PDF.

[5] Zhang C., Wang X., Kinoshita J.-I., Zhao B., Toko T., Kimura Y., Matsumoto K. (2007). Effects of KTP Laser Irradiation, Diode Laser, and LED on Tooth Bleaching: A Comparative Study. Photomed. Laser Surg..

[6] Gurgan S., Cakir F.Y., Yazici E. (2010). Different light-activated in-office bleaching systems: A clinical evaluation. Lasers Med. Sci..

[7] Heiskanen V., Hamblin M.R. (2018). Photobiomodulation: Lasers: Vs. light emitting diodes?. Photochem. Photobiol. Sci..

[8] Buchalla W., Attin T. (2007). External bleaching therapy with activation by heat, light or laser—A systematic review. Dent. Mater..

[9] Lin M., Xu F., Lu T.J., Bai B.F. (2010). A review of heat transfer in human tooth-Experimental characterization and mathematical modeling. Dent. Mater..

[10] Niu L., Dong S.J., Kong T.T., Wang R., Zou R., Liu Q. (2016). Da Heat transfer behavior across the dentino-enamel junction in the human tooth. PLoS ONE.

[11] Odor T.M., Watson T.F., Pitt Ford T.R., Mcdonald F. (1996). Pattern of transmission of laser light in teeth. Int. Endod. J..

[12] Lin M., Liu Q.D., Kim T., Xu F., Bai B.F., Lu T.J. (2010). A new method for characterization of thermal properties of human enamel and dentine: Influence of microstructure. Infrared Phys. Technol..

[13] Zach L., Cohen G. (1965). Pulp response to externally applied heat. Oral Surg. Oral Med. Oral Pathol..

[14] Eriksson A.R., Albrektsson T. (1983). Temperature threshold levels for heat-induced bone tissue injury: A vital-microscopic study in the rabbit. J. Prosthet. Dent..

[15] Meire M.A., Poelman D., De Moor R.J. (2014). Optical properties of root canal irrigants in the 300-3000-nm wavelength region. Lasers Med. Sci..

[16] Al-Karadaghi T.S., Al-Saedi A.A., Al-Maliky M.A., Mahmood A.S. (2016). The effect of bleaching gel and (940 nm and 980 nm) diode lasers photoactivation on intrapulpal temperature and teeth whitening efficiency. Aust. Endod. J..

[17] Heydent Next Safety Data Sheet According to 1907/2006/EC, Article 31. https://www.heydent.com/fileadmin/Heydent/MSDS/MSDS_NEXT.pdf.

[18] SDI Pola Office + Safety Data Sheet According to GHS. https://www.sdi.com.au/wp-content/uploads/SDS/SDS_CR_CZ/Pola_Office_+_DS_EN.pdf.

[19] Whitesmile Safety Data Sheet According to 1907/2006/EC. https://www.whitesmile.de/wp-content/uploads/WHITEsmile_MSDS_13_05_2015_POWER_LIGHT-WHITENING_GB.pdf.

[20] Biolase Laserwhite20 Safety Data Sheet. https://www.msdsdigital.com/sites/default/files/msds_record_database/Laser%20white%20.pdf.

[21] IDS BlancOne Safety Data Sheet. https://www.blancone.eu/wp-content/uploads/2017/09/MSDS-BlancOne-ULTRA-V3.-0-EN.pdf.

[22] Ultradent Opalescence Boost Safety Data Sheet According to Regulation (EC) No. 453/2010 Dental Use. https://intl.ultradent.com/eu/MSDS/Opalescence%20Boost%20(Part%201%20of%202%20-%20Bleaching%20Gel).pdf.

[23] Goharkhay K., Schoop U., Wernisch J., Hartl S., De Moor R., Moritz A. (2009). Frequency doubled neodymium:yttrium-aluminum-garnet and diode laser-activated power bleaching-pH, environmental scanning electron microscopy, and colorimetric in vitro evaluations. Lasers Med. Sci..

[24] Batista G.R., Barcellos D.C., Borges A.B., Torres C.R.G. (2011). Analysis of the Pulp Chamber Temperature of Teeth Submitted to Light Activation with and without Bleaching Gel. World J. Dent..

[25] Goldstein R., Garber D. (1995). Complete Dental Bleaching.

[26] De Moor R.J.G., Verheyen J., Verheyen P., Diachuk A., Meire M.A., De Coster P.J., De Bruyne M., Keulemans F. (2015). Laser teeth bleaching: Evaluation of eventual side effects on enamel and the pulp and the efficiency in vitro and in vivo. Sci. World J..

[27] Mó O., Yáñez M., Eckert-Maksić M., Maksić Z.B., Alkorta I., Elguero J. (2005). Periodic trends in bond dissociation energies. A theoretical study. J. Phys. Chem. A.

[28] Li Y., Greenwall L. (2013). Safety issues of tooth whitening using peroxide-based materials. Br. Dent. J..

[29] Khan I., Tang E., Arany P. (2015). Molecular pathway of near-infrared laser phototoxicity involves ATF-4 orchestrated ER stress. Sci. Rep..

[30] Caviedes-Bucheli J., Ariza-García G., Restrepo-Méndez S., Ríos-Osorio N., Lombana N., Muñoz H.R. (2008). The Effect of Tooth Bleaching on Substance P Expression in Human Dental Pulp. J. Endod..

[31] Berkovitz B., Boyde A., Frank R., Hohling H., Moxham B., Nalbandian J., Tonge C. (1989). Handbook of Microscopic Anatomy Vol.6 Teeth.

[32] Seka W., Fried D., Featherstone J.D., Borzillary S.F. (1995). Light deposition in dental hard tissue and simulated thermal response. J. Dent. Res..

[33] Fried D., Glena R.E., Featherstone J.D.B., Seka W. (1995). Nature of light scattering in dental enamel and dentin at visible and near-infrared wavelengths. Appl. Opt..

[34] Kreisler M., Al-Haj H., D’Hoedt B. (2002). Intrapulpal temperature changes during root surface irradiation with an 809-nm GaAlAs laser. Oral Surg. Oral Med. Oral Pathol. Oral Radiol. Endod..

[35] Pothiawala S., Kilmer S., Ibrahimi O., Nouri K. (2014). Basic Principles of Lasers: Interactions between Lasers and Tissue. Handbook of Lasers in Dermatology.

[36] Murphy M.J., Torstensson P.A. (2014). Thermal relaxation times: An outdated concept in photothermal treatments. Lasers Med. Sci..

[37] Parker S. (2007). Verifiable CPD paper: Laser-tissue interaction. Br. Dent. J..

[38] Selting W., Coluzzi D.J., Parker S.P.A. (2017). Laser Operating Parameters for Hard and Soft Tissue, Surgical and PBM Management. Lasers in Dentistry—Current Concepts.

[39] International Organization for Standardization (2018). Optics and Photonics—Lasers and Laser-Related Equipment—Test Methods for Laser Beam Power (Energy) Density Distribution.

[40] Sari T., Celik G., Usumez A. (2015). Temperature rise in pulp and gel during laser-activated bleaching: In vitro. Lasers Med. Sci..

[41] Kijsamanmith K., Vongsavan N., Matthews B. (2018). Pulpal blood flow recorded from exposed dentine with a laser Doppler flow meter using red or infrared light. Arch. Oral Biol..

[42] Yu C., Abbott P. (2007). An overview of the dental pulp: Its functions and responses to injury. Aust. Dent. J..

[43] Markowitz K. (2010). Pretty painful: Why does tooth bleaching hurt?. Med. Hypotheses.

[44] Scribante A., Poggio C., Gallo S., Riva P., Cuocci A., Carbone M., Arciola C., Colombo M. (2020). In Vitro Re-Hardening of Bleached Enamel Using Mineralizing Pastes: Toward Preventing Bacterial Colonization. Materials.

[45] Epple M., Meyer F., Enax J. (2019). A critical review of modern concepts for teeth whitening. Dent. J..

[46] Attin T., Schmidlin P.R., Wegehaupt F., Wiegand A. (2009). Influence of study design on the impact of bleaching agents on dental enamel microhardness: A review. Dent. Mater..

[47] Anagnostaki E., Luk K., Coluzzi D.J., Parker S.P.A. (2017). Impact of Laser Dentistry in Management of Color in Aesthetic Zone. Lasers in Dentistry—Current Concepts.

[48] Vano M., Derchi G., Barone A., Genovesi A., Covani U. (2015). Tooth bleaching with hydrogen peroxide and nano-hydroxyapatite: A 9-month follow-up randomized clinical trial. Int. J. Dent. Hyg..

[49] Usai P., Campanella V., Sotgiu G., Spano G., Pinna R., Eramo S., Saderi L., Garcia-Godoy F., Derchi G., Mastandrea G. (2019). Effectiveness of Calcium Phosphate Desensitising Agents in Dental Hypersensitivity Over 24 Weeks of Clinical Evaluation. Nanomaterials.

[50] Moosavi H., Arjmand N., Ahrari F., Zakeri M., Maleknejad F. (2016). Effect of low-level laser therapy on tooth sensitivity induced by in-office bleaching. Lasers Med. Sci..

[51] De Paula B., Alencar C., Ortiz M., Couto R., Araújo J., Silva C. (2019). Effect of photobiomodulation with low-level laser therapy combined with potassium nitrate on controlling post-bleaching tooth sensitivity: Clinical, randomized, controlled, double-blind, and split-mouth study. Clin. Oral Investig..

[52] Luong M.N., Otsuki M., Shimada Y., Ei T.Z., Sumi Y., Tagami J. (2019). Effect of lights with various wavelengths on bleaching by 30% hydrogen peroxide. Lasers Med. Sci..

[53] Goldberg M., Grootveld M., Lynch E. (2010). Undesirable and adverse effects of tooth-whitening products: A review. Clin. Oral Investig..

[54] Rodríguez-Martínez J., Valiente M., Sánchez-Martín M. (2019). Tooth whitening: From the established treatments to novel approaches to prevent side effects. J. Esthet. Restor. Dent..

